# Multi-Responsive Polysiloxane/Poly(*N*-isopropylacrylamide) Interpenetrating Networks Containing Urea and Thiourea Groups

**DOI:** 10.3390/polym12051175

**Published:** 2020-05-20

**Authors:** Junta Sano, Shigeki Habaue

**Affiliations:** Department of Applied Chemistry, College of Engineering, Chubu University, 1200 Matsumoto-cho, Kasugai, Aichi 487-8501, Japan; tk19801-6197@sti.chubu.ac.jp

**Keywords:** polysiloxane, poly(*N*-isopropylacrylamide), interpenetrating polymer networks, swelling property, anion sensing

## Abstract

Novel interpenetrating polymer networks (IPNs) were synthesized from *N*-isopropylacrylamide (NIPAM) and polysiloxanes containing a urea or thiourea side group, in addition to the silanol residue, through two reactions, such as the radical gelation of NIPAM and the condensation of silanols to form a siloxane linkage. The obtained IPNs showed a typical temperature-responsive volume change in water based on the constructed poly-NIPAM gel component. In addition, the characteristic color and volume changes responding to chemical stimuli, such as acetate and/or fluoride ions, based on the introduced urea and thiourea groups onto the polysiloxane gel were observed in *N,N*-dimethylformamide.

## 1. Introduction

Interpenetrating polymer networks (IPNs) are a gel with an interesting structure, which consists of two polymeric components as an entangled network structure without covalent bonds crosslinking each other. Therefore, the IPNs have attracted much attention as new materials with mechanical, stimuli-responsive, and biophysical properties for various applications that include drug delivery systems, membranes, sensors, and biomedical uses [1,2,3,4,5,6]. The IPNs are generally classified into two groups based on their framework, i.e., semi- and full IPNs. The synthesis of the latter is more restricted than the former because full IPNs are synthesized in a sequential or simultaneous manner, that is, two reactions of the precursors constructing the respective networks must be performed in a stepwise fashion or at the same time without interfering with each other, respectively. 

Stimuli-responsive gels are typically capable of controlling the volume change in response to various external conditions, such as temperature, pH, and chemicals. In particular, the IPNs can be applied to dual-responsive materials, which respond to two factors. Among such smart IPN gels, temperature- and pH-responsive hydrogels have been extensively studied because these two factors can be easily controlled in an aqueous phase [7,8,9,10,11]. Thus, the smart IPNs that multiply responding to the external stimuli in various media are still limited mainly due to difficulty in synthesizing the designed IPN structure. 

Recently, we demonstrated the synthesis of a full IPN composed of polysiloxane and acrylamide gels through the novel simultaneous method, where both the radical gelation of acrylamides in the presence of a crosslinking agent and the condensation reaction between silanols on the polysiloxane proceeded simultaneously [12]. The polysiloxane with a silicon–oxygen backbone is one of the most important classes of inorganic polymers and shows a typical hydrophobic property, whereas polyacrylamide, such as poly(*N*-isopropylacrylamide) (PNIPAM), is known as a hydrophilic and water-soluble polymer with a thermo-responsive lower critical solution temperature (LCST) of approximately 32 °C. Therefore, the synthesized IPN could have the characteristics of an organic–inorganic hybrid-type material. 

**Scheme 1:**
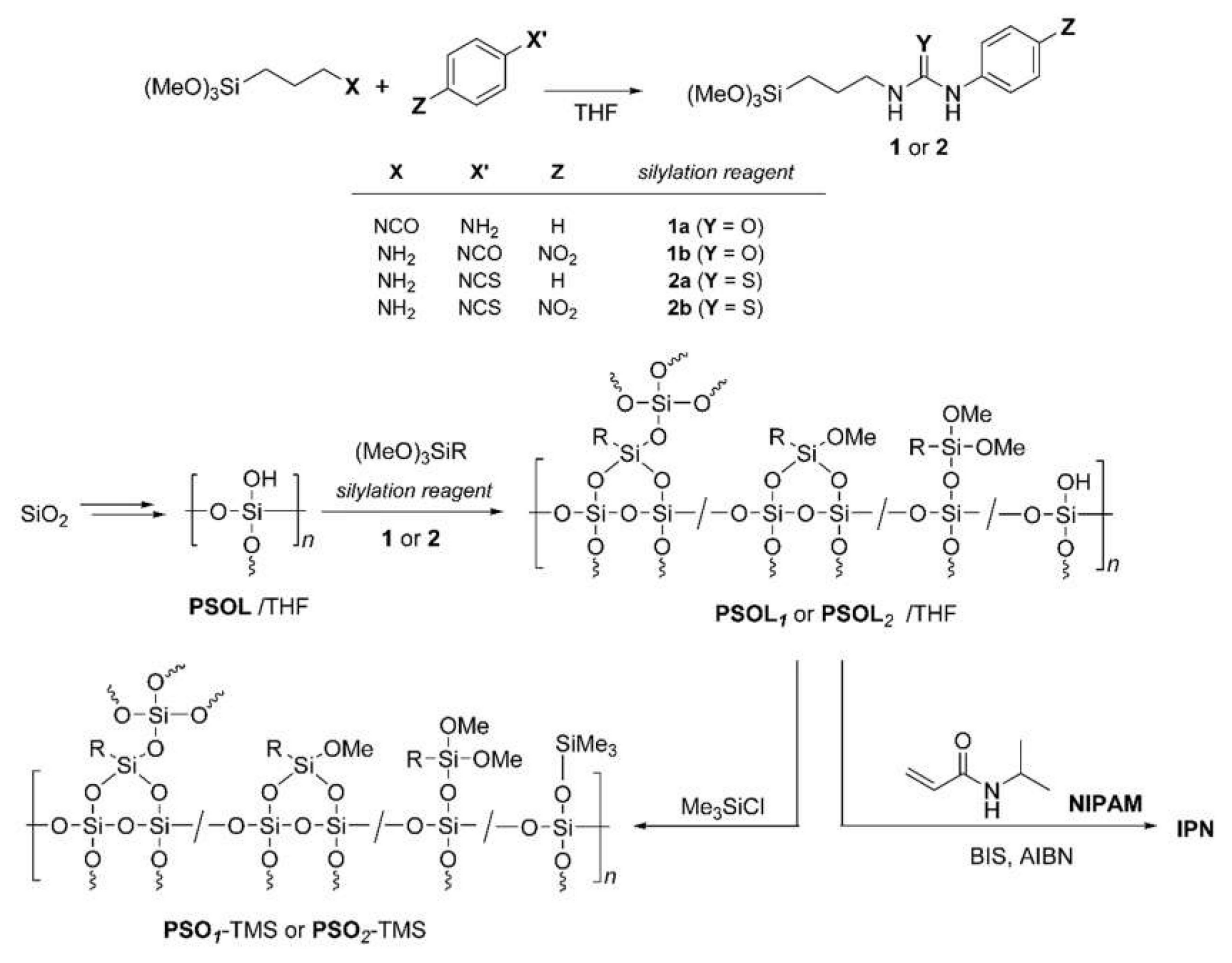
Interpenetrating polymer network (IPN) synthesis with polysiloxanes and *N*-isopropylacrylamide (NIPAM).

Among the present synthetic routes, it is known that polysiloxane can be prepared from silica gel or water glass [13,14,15,16,17]. The precursor polymer containing the silanol residue is prepared simply by the partial silylation reaction of silanols on a polymeric silicic acid ([SiO(OH)_2_]_n_, **PSOL**), which is derived from silica gel, with silylation reagents, such as trialkoxysilanes [RSi(OR’)_3_] (Scheme 1). Various silylation reagents can be employed for this procedure, then functionalized polysiloxanes can be easily produced with marked contrast to the conventional poly(dialkylsiloxane) with respect to the design ability. In fact, the polysiloxanes bearing the aromatic and aliphatic side groups, in addition to the silanol, were used for the reported IPN synthesis [12]. 

On the other hand, it is known that simple thiourea-based receptors exclusively bind acetate and fluoride anions through hydrogen bond formation along with a color change [18,19,20,21,22]. For example, 1,3-bis(*p*-nitrophenyl)thiourea showed a chromogenic response to the chemical stimuli of the acetate ion in acetonitrile [18]. The polyacrylate gels containing a *p*-nitrophenylthiourea side chain also showed a selective response with both volume and color changes toward the acetate and fluoride anions in tetrahydrofuran (THF) [22]. 

In this study, novel IPNs composed of PNIPAM and polysiloxanes bearing urea and thiourea side groups were synthesized, and their behaviors to the stimuli, such as temperature and anion species, were investigated. The PNIPAM gel demonstrated a temperature-responsive volume change based on the volume phase transition temperature (VPTT) in water, while the thiourea chromophore on the silicone polymer has the function of responding to the anions of tetrabutylammonium salts in an organic solvent. Accordingly, IPNs could work as novel hybrid-type multi-responsive materials.

## 2. Materials and Methods 

### 2.1. Measurements

The ^1^H NMR spectra were obtained using a JEOL JNM-ECS400 (400 MHz for ^1^H) spectrometer (JEOL, Akishima, Japan). Size-exclusion chromatography (SEC) was conducted using a LC-20AD (Shimadzu, Kyoto, Japan) system equipped with column oven (CTO-20A), UV/Vis (SPD-20A), RI (RID-20A) detectors, and TSKgel G3000H and G7000H columns (Tosoh, Tokyo, Japan) for THF connected in series. Calibration was carried out using polystyrene standards. Infrared (FT-IR) spectra were recorded using a Shimadzu IR Affinity-1S spectrometer.

### 2.2. Materials

The silica gel (spherical (particle size: 63–210 μm), neutral) (Kanto, Tokyo, Japan), silylation reagents, such as 3-isocyanatopropyl(trimethoxy)silane, 3-aminopropyl(trimethoxy)silane, and chloro(trimethyl)silane (TMSCl) (TCI, Tokyo, Japan), and the other chemicals for the in situ preparation of urea and thiourea compounds, such as aniline (Wako, Osaka, Japan), 4-nitrophenyl isocyanate, phenyl isothiocyanate, and 4-nitrophenyl isothiocyanate (TCI), were used as received. A vinyl monomer, NIPAM, and a crosslinker, *N,N′*-methylenebisacrylamide (BIS; Wako, Osaka, Japan), were utilized after purification by recrystallization. A solvent, THF, and a radical initiator, azobisisobutyronitrile (AIBN; Wako, Osaka, Japan), were used for IPN gel synthesis. Various tetrabutylammonium salts (TCI) were employed as anions for the chemical stimuli. 

### 2.3. IPN Synthesis

Silylation reagents bearing urea (**1a** and **1b**) and thiourea groups (**2a** and **2b**) were generated in situ through a reaction between the corresponding amines and compounds with the isocyanate or isothiocyanate group (1/1, mol/mol) in THF (1.0 M) for 2 h at room temperature under a N_2_ atmosphere, as shown in Scheme 1. A THF solution of the silicone polymer containing a silanol residue, such as **PSOL*_1_*** and **PSOL*_2_***, which were prepared from **PSOL** with the above-mentioned silylation reagents, was used without isolation, as previously reported [12,16,17]. 

To a solution of **PSOL*_1_*** or **PSOL*_2_*** in a perfluoroalkoxy (PFA) resin test tube, NIPAM (0.77 g, 6.8 mmol), BIS, and AIBN ((NIPAM)/(BIS)/(AIBN) = 100/4/1) were introduced. After gelation for 24 h at 60 °C under a N_2_ atmosphere, the obtained gel was immersed in THF for 2 days, then in distilled water for 2 days. It was further dried overnight under reduced pressure at 80 °C. 

The swelling degree of the obtained gels was estimated using the typical gravimetric method [22,23]. The gels were immersed in an excess amount of distilled water, *N,N*-dimethylformamide (DMF), or a DMF solution of a tetrabutylammonium salt for 24 h at the appropriate temperature, then the swollen gels were removed from the medium and weighed. The degree of swelling (*Q*) was calculated according to the following equation:

*Q* = (*m*_w_ − *m*_d_)/*m*_d_, (1)

where *m*_w_ and *m*_d_ are the weights of the wet and dried samples, respectively.

## 3. Results and Discussion

### 3.1. Structure of Silicone Polymers

**Figure 1:**
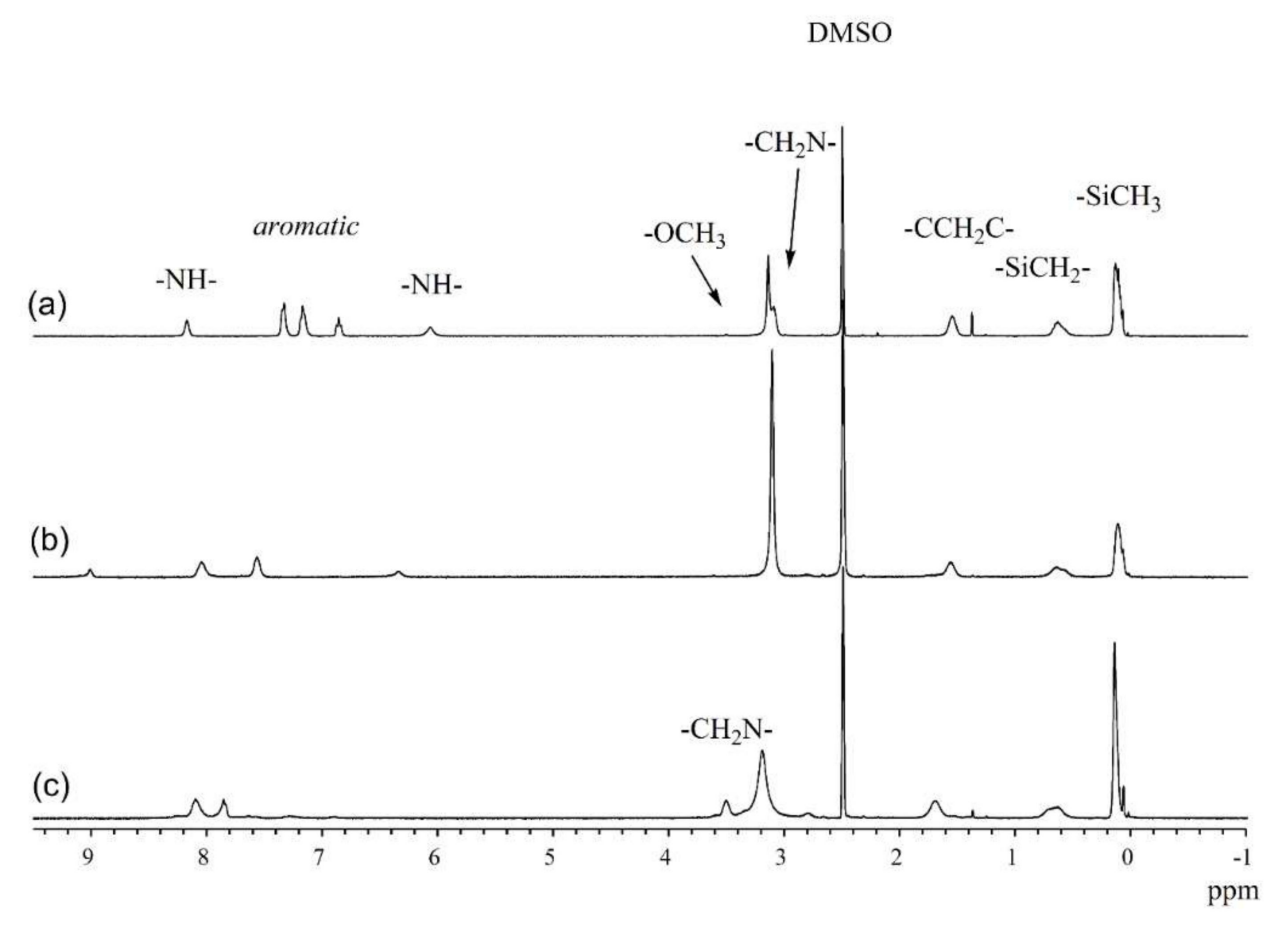
^1^H NMR spectra of (**a**) **PSO*_1a(0.1)_***-TMS (Table 1, run 1), (**b**) **PSO*_1b(0.1)_***-TMS (run 4), and (**c**) **PSO*_2b(0.1)_***-TMS (run 8) (DMSO-*d*_6_, 80 °C).

To estimate the ratio of silanol on the prepared **PSOL*_1_*** and **PSOL*_2_***, the reactive silanol residue was further silylated with an excess amount of TMSCl to isolate the polymers, **PSO*_1_***-TMS and **PSO*_2_***-TMS (Scheme 1). For instance, 0.05 g of **PSO*_1a(0.1)_***-TMS (*M*_w_ = 3.2 × 10^3^, *M*_w_/*M*_n_ = 2.2) synthesized with 0.1 equiv. of **1a** against the used SiO_2_ (0.20 g) was recovered as the insoluble part in a mixture of methanol and water (2/1). Figure 1 demonstrates the ^1^H NMR spectra of the obtained polymers. The signals are assigned as shown in the figure, clearly indicating that the polymers have the urea and thiourea moieties as a side group. For example, the unit ratio of **PSO*_1a(0.1)_***-TMS was also evaluated to be PhNHCONH(CH_2_)_3_/MeO/TMS = 59/2/39, where the content of the TMS group corresponds to that of the silanol residue in the **PSOL*_1a(0.1)_***. In every case, the condensation between the silanol and methoxysilyl groups of the silylation reagents effectively proceeded on the basis of the observation that the methoxy group in the obtained polymer was a small amount (3% or less). Accordingly, in the original **PSOL*_1a(0.1)_***, 18% of the silanol remains after silylation with **1a**, assuming that the RSi(OMe)_2_- unit is negligible [12]. The evaluated ratios of the silanol residue for **PSOL*_1_*** and **PSOL*_2_*** are listed in Table 1. The ratios decreased with the increasing amount of the silylation reagents. 

### 3.2. Synthesis of IPNs

**Figure 2:**
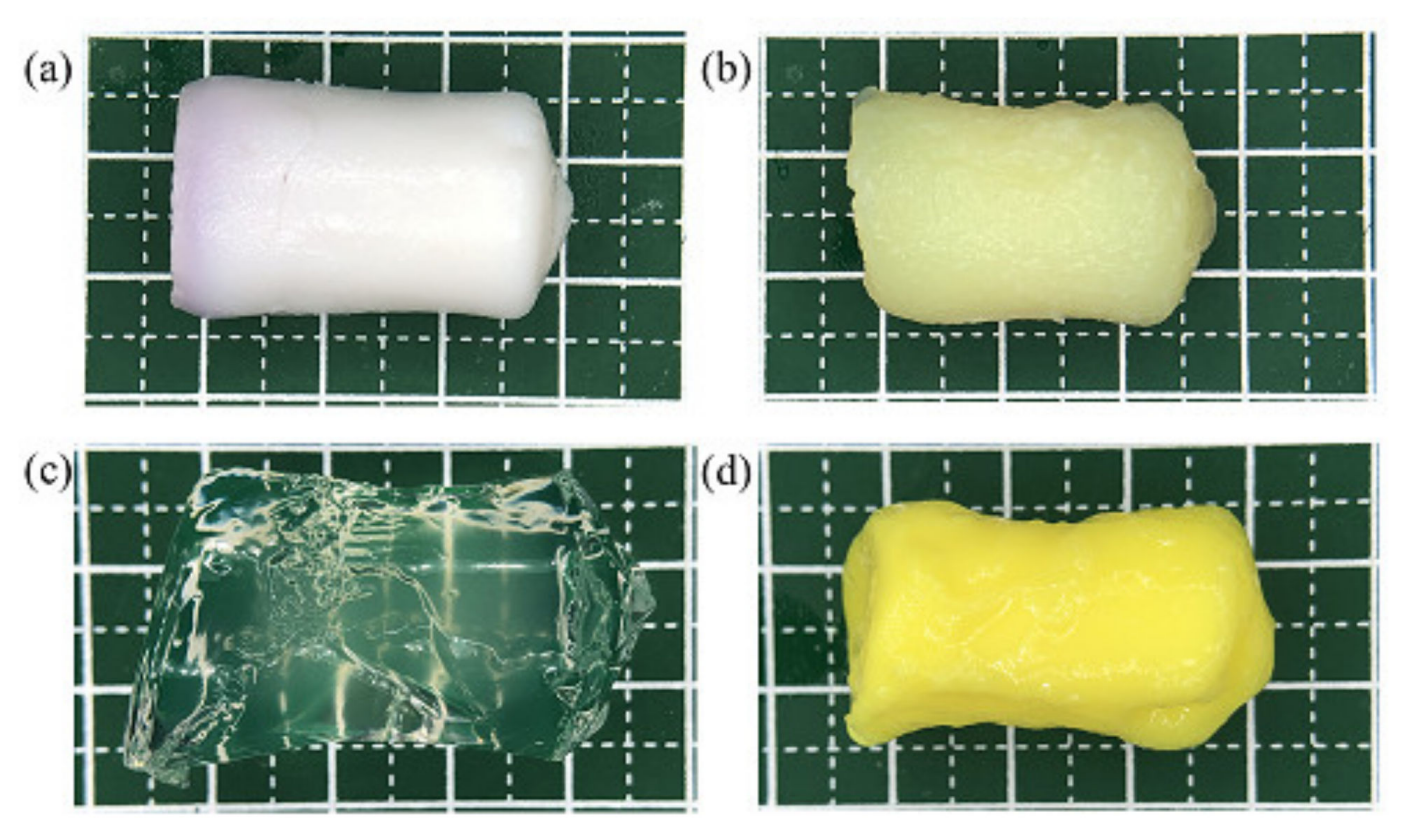
Photo images of the obtained IPN organogels in THF; (**a**) **IPN*_1a(0.2)_***, (**b**) **IPN*_1b(0.2)_***, (**c**) **IPN*_2a(0.2)_***, and (**d**) **IPN*_2b(0.2)_***.

Table 2 summarizes the results of the IPN synthesis using the prepared silicone polymers, NIPAM and BIS, together with those of the preparation of the PNIPAM gel (**G*_NIPAM_***, run 1). The gelation effectively proceeded to produce homogeneous organogels, whose color was white and almost clear when prepared with **PSOL*_1a_*** and **PSOL*_2a_***, whereas the organogels obtained with **PSOL*_1b_*** and **PSOL*_2b_*** were slightly yellow and yellow, respectively (Figure 2). They were isolated in a good yield as a hard gel after washing with THF and water, followed by vacuum drying. For example, the gelation of **PSOL*_1a(0.1)_*** and NIPAM in the presence of BIS produced 0.75 g of a THF- and water-insoluble product, **IPN*_1a(0.1)_*** (run 2). 

When a THF solution of the prepared polysiloxane containing the silanol residue was left at 60 °C in the absence of a vinyl monomer, a crosslinker, and a radical initiator, the condensation of the silanols, a crosslinking reaction, occurred to produce an insoluble gel, as previously reported [12]. The model semi-IPN was also prepared by the gelation of NIPAM (0.67 g, (NIPAM)/(BIS)/(AIBN) = 100/4/1) in the presence of the isolated polysiloxane, **PSO*_1a(0.4)_***-TMS (0.30 g, *M*_w_ = 3.1 × 10^3^, *M*_w_/*M*_n_ = 2.0), in THF with a yield of 0.72 g after sufficiently washing with THF and water. The obtained model gel as well as G*_NIPAM_* (run 1) were brittle; therefore, the gels synthesized using the simultaneous gelation method (runs 2–10) showed an improved mechanical property, which is one of the typical characteristics observed for full IPNs [1,2]. These results suggest that the gel structure of the polysiloxane constructed during the gelation significantly affects the properties of the IPNs.

**Figure 3:**
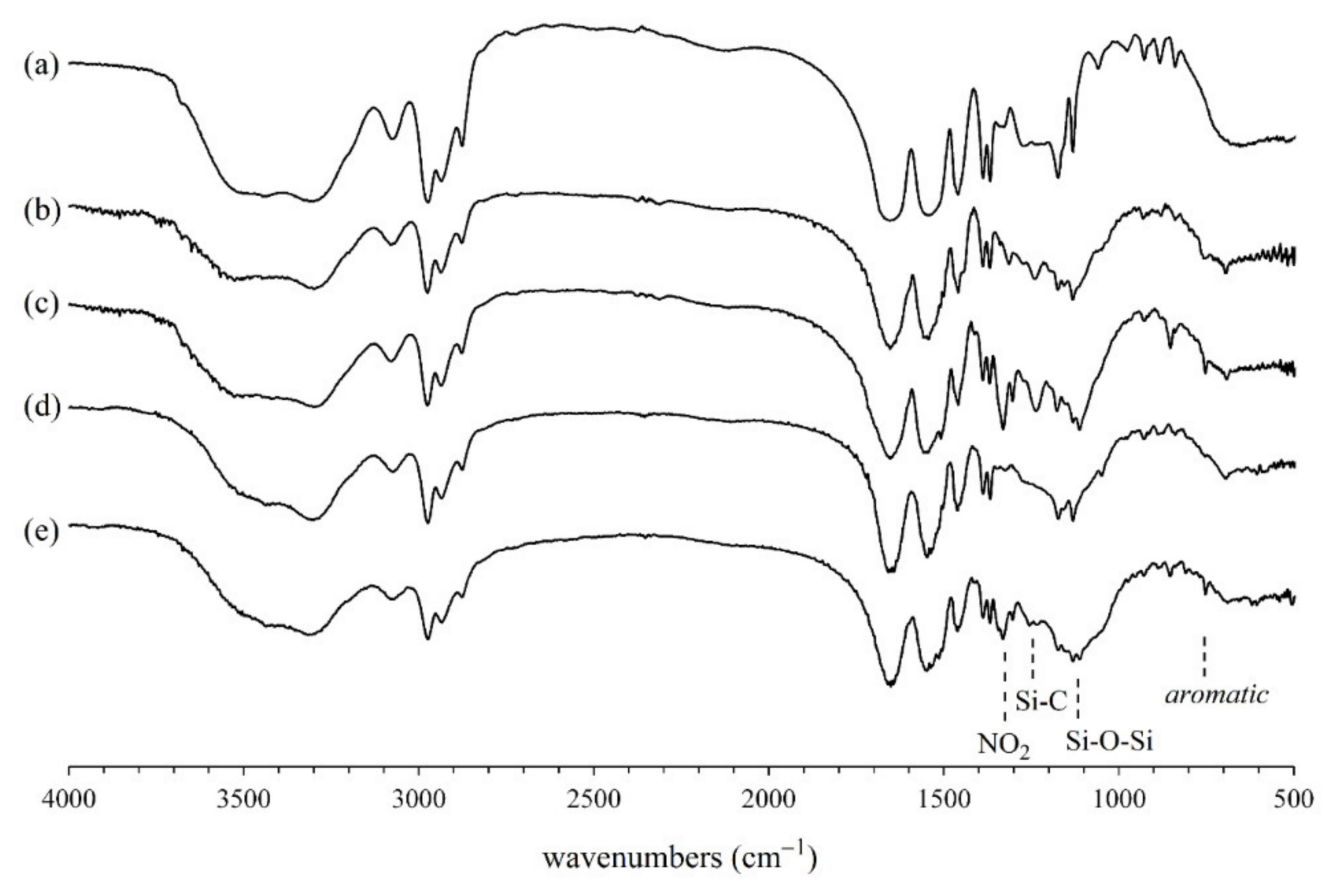
IR spectra of (**a**) **G*_NIPAM_*** (Table 2, run 1), (**b**) **IPN*_1a(0.2)_*** (run 3), (**c**) **IPN*_1b(0.2)_*** (run 6), (**d**) **IPN*_2a(0.2)_*** (run 8), and (**e**) **IPN*_2b(0.2)_*** (run 10) (KBr).

The FT-IR spectra of the gels, such as **IPN*_1a(0.2)_***, **IPN*_1b(0.2)_***, **IPN*_2a(0.2)_,*** and **IPN*_2b(0.2)_*** (Table 2, runs 3, 6, 8, and 10), are shown in Figure 3 together with those of **G*_NIPAM_*** (run 1). The characteristic absorptions based on the Si–O and Si–C bonds for the silicone polymer and the nitrophenyl group in the side chain, in addition to those of PNIPAM, were clearly observed. These results indicate that the obtained gels are composed of the silicone polymer and PNIPAM gels and have an IPN structure.

### 3.3. Swelling Properties of the Obtained IPNs

**Figure 4:**
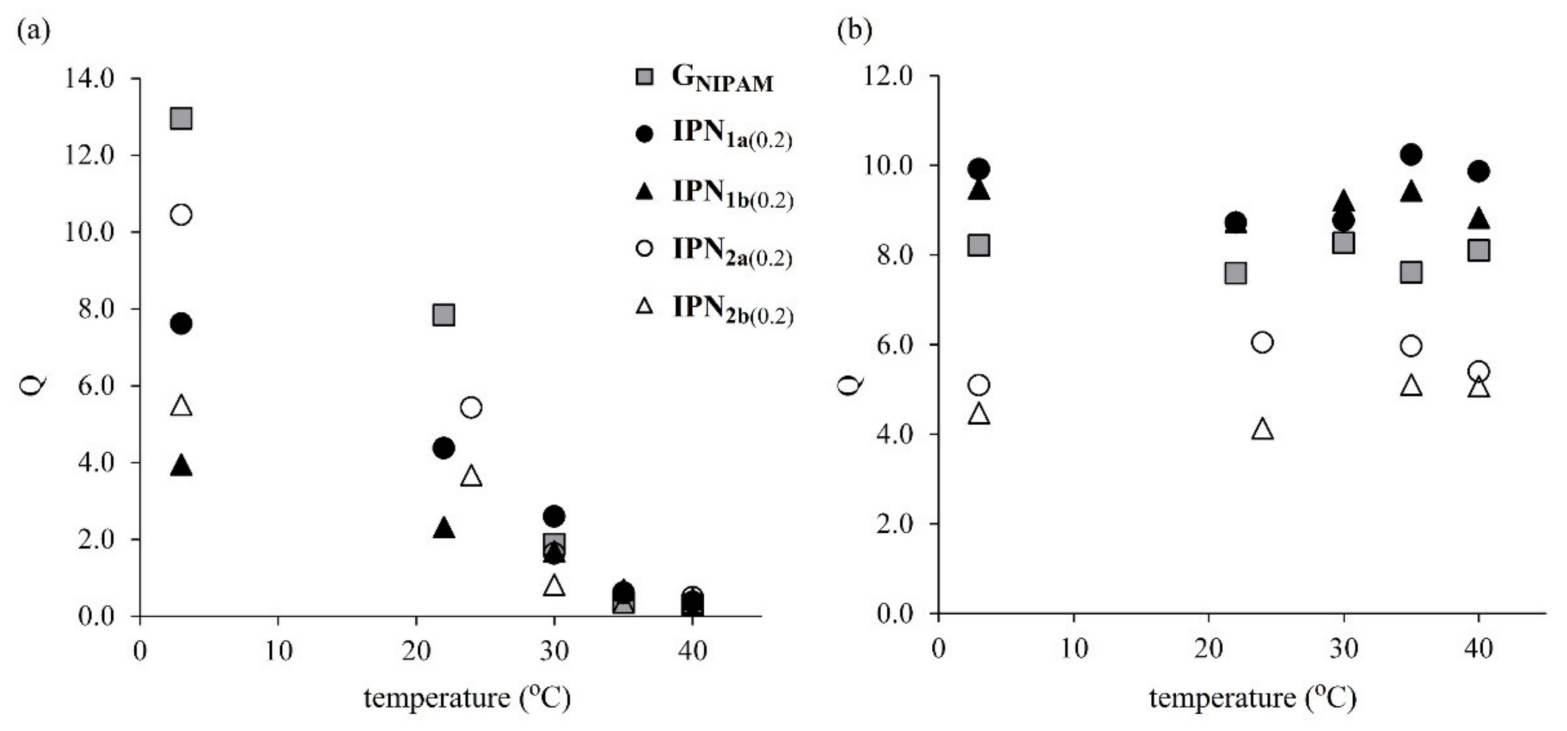
Plots of swelling degree (*Q*) of **G*_NIPAM_*** (Table 2, run 1) (■), **IPN*_1a(0.2)_*** (run 3) (●), **IPN*_1b(0.2)_*** (run 6) (▲), **IPN*_2a(0.2)_*** (run 8) (○), and **IPN*_2b(0.2)_*** (run 10) (△) (**a**) in water and (**b**) in *N,N*-dimethylformamide (DMF).

The observed swelling degrees (*Q*) for the obtained IPNs at 3 °C in water are also listed in Table 2, and their swelling behaviors, which depend on the temperature, are depicted in Figure 4a. The PNIPAM hydrogel shows a *Q* value of 13.0 at 3 °C (run 1) and a drastic decrease with the increase in temperature based on the VPTT, as shown in the figure (■). The *Q* values measured for the obtained IPNs were lower than that observed for **G*_NIPAM_***. These results may be due to the hydrophobic property of the introduced silicone polymer component, as well as the constructed IPN structure. The IPNs containing a nitro group, such as **IPN*_1b_*** and **IPN*_2b_***, showed a tendency to have lower values of *Q* (▲ and △) than those observed for the gels without a nitro group (● and ○). Meanwhile, every IPN demonstrated a similar behavior versus temperature to that observed for **G*_NIPAM_*** based on the VPTT of the PNIPAM gel component, therefore indicating that the synthesized IPNs have a function response to temperature with a volume change in water. 

In an organic solvent, such as DMF, in contrast, both components, namely, the silicone polymer and PNIPAM networks, can swell. Accordingly, the IPNs showed almost constant values versus the temperature, which were similar to that observed for **G*_NIPAM_*** (■), as shown in Figure 4b. The IPNs containing a thiourea group on the silicone polymer, such as **IPN*_2a_*** and **IPN*_2b_***, showed lower values of *Q* (○ and △), while those observed for the IPNs having urea groups, such as **IPN*_1a_*** and **IPN*_1b_***, (● and ▲) were higher than that of **G*_NIPAM_***.

### 3.4. Behaviors of the Obtained IPNs to Anion Stimuli

**Figure 5:**
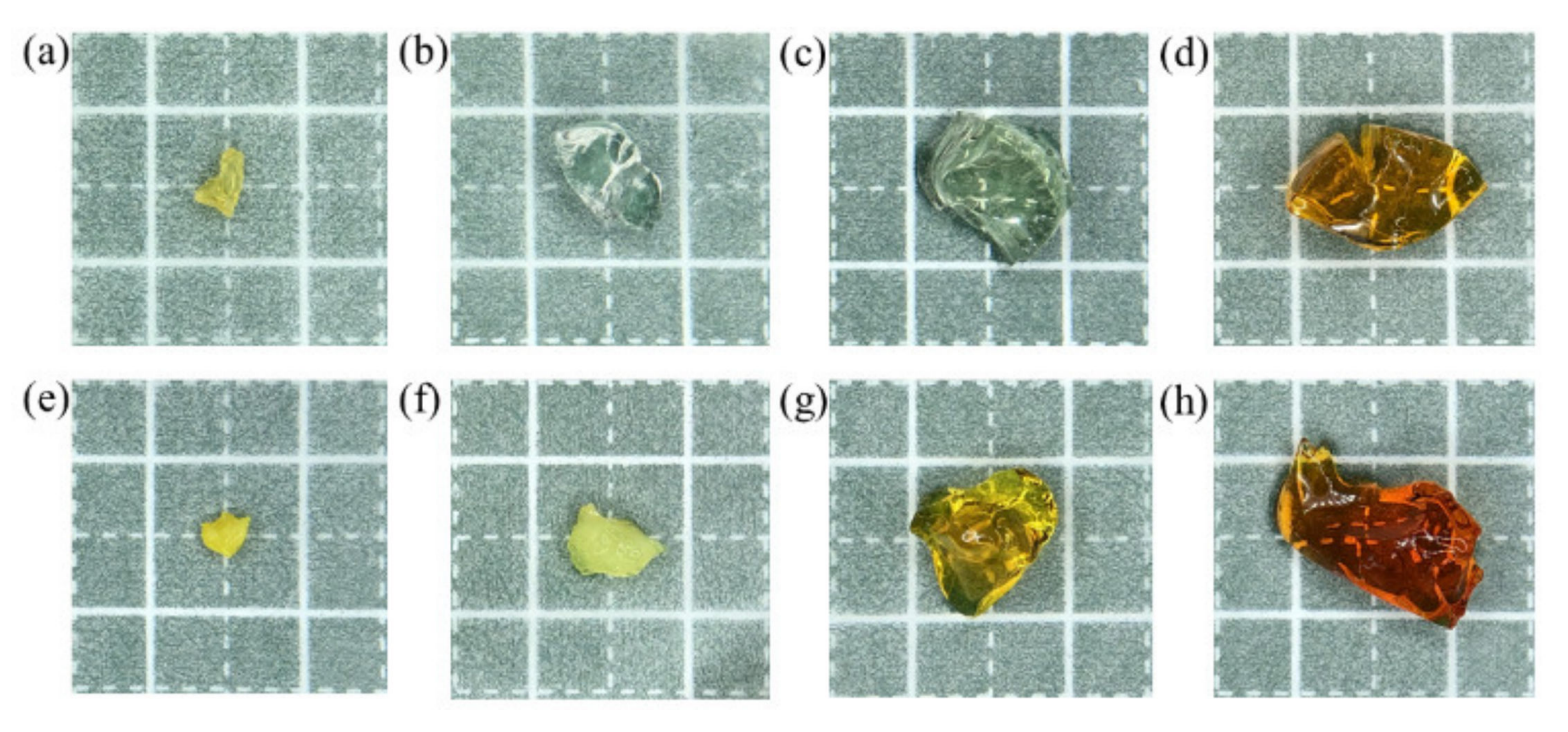
Photo images of **IPN*_1b(0.2)_*** (**a**–**d**) and **IPN*_2b(0.2)_*** (**e**–**h**); the dried gels (**a**,**e**), the organogels in the absence of TBAX (**b**,**f**), in the presence of acetate (**c**,**g**) and fluoride ions (**d**,**h**) in DMF.

The swelling experiments in the presence of various tetrabutylammonium salts (TBAX, 20 mM) in DMF at 25 °C were then carried out, and the results are listed in Table 3. The dried gels, such as **IPN*_1b_*** and **IPN*_2b_***, containing a *p*-nitrophenyl chromophore, turned yellow-orange and red, respectively, by immersing in the fluoride ion solution, while **IPN*_2a_*** did not change color to give a clear organogel. When the DMF solution of the acetate ion was used, the color change hardly occurred for **IPN*_1b_***, as well as **IPN*_2a_***, whereas the swelled **IPN*_2b_*** showed a yellow-orange color change (Figure 5). The effects of the other ions, such as chloride, bromide, etc., were almost negligible for both volume and color changes of the IPNs. Therefore, the synthesized **IPN*_1b_*** and **IPN*_2b_*** demonstrated a specific color change response to the anion species as external stimuli due to the complex formation of *p*-nitrophenylurea and *p*-nitrophenylthiourea with acetate and/or fluoride ions [18,19,20,21,22]. 

**Figure 6:**
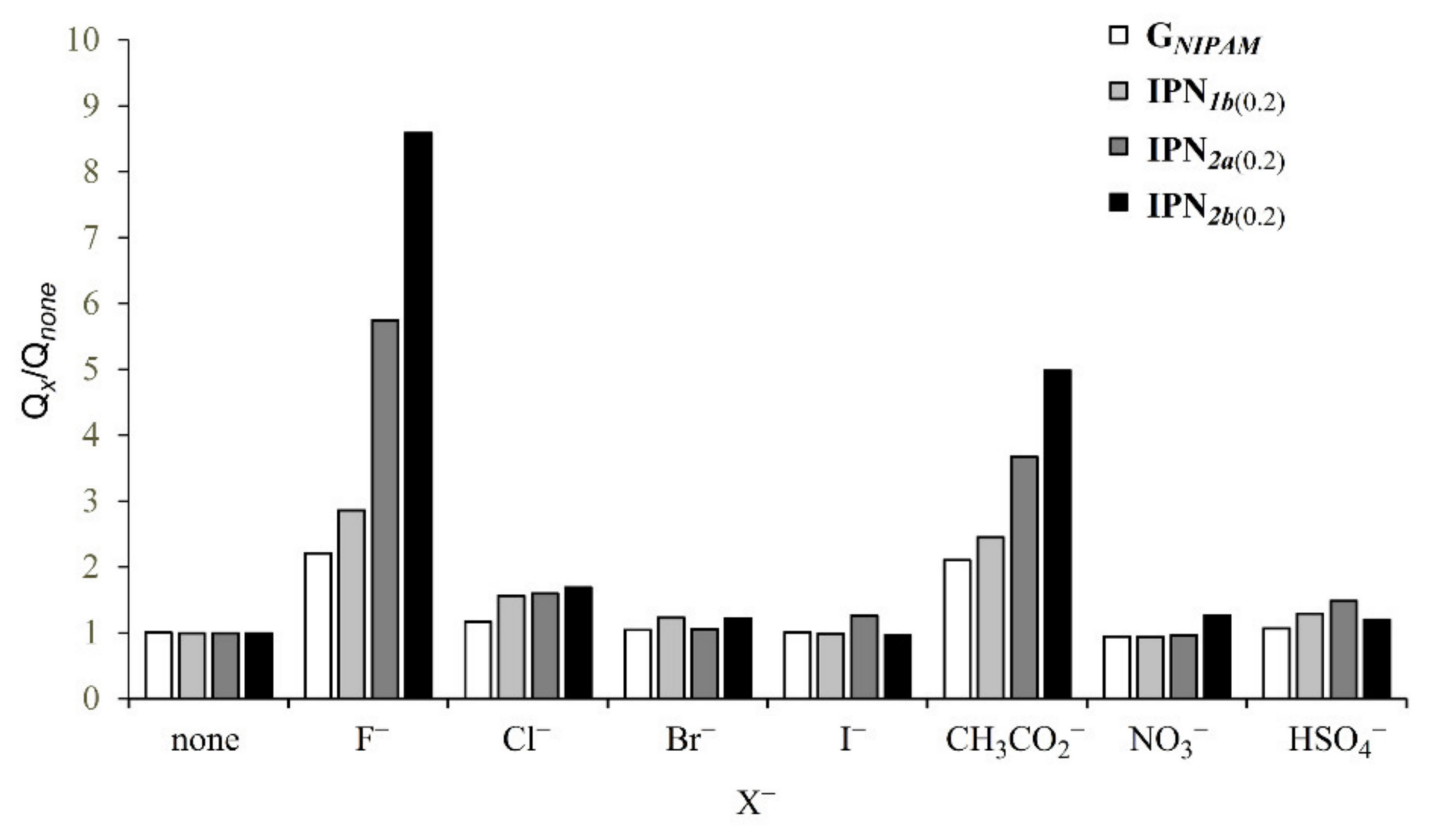
Relative swelling degree ratio (*Q*_x_/*Q_none_*) of **G*_NIPAM_*** (Table 2, run 1), **IPN*_1b(0.2)_*** (run 6), **IPN*_2a(0.2)_*** (run 8), and **IPN*_2b(0.2)_*** (run 10) in the presence of TBAX (20 mM) in DMF (25 °C).

In addition, the *Q* values were significantly increased in the presence of the acetate and fluoride ions. Particularly, the value estimated for **IPN*_2b(0.2)_*** in the presence of the latter reached approximately 36, which is much higher than that observed in the absence of the anion species, *Q* = 4.2, as well as that of **G*_NIPAM_***. The swelling behaviors of the obtained IPNs in the presence of various TBAXs were evaluated by the relative swelling ratios, *Q*_x_/*Q*_none_, where *Q*_x_ and *Q*_none_ are the swelling degrees in the presence and absence of X^−^, respectively, as shown in Figure 6. The results indicated that the combination of the **IPN*_2a_*** and **IPN*_2b_*** with the acetate and fluoride ions effectively caused a selective volume change in the swelling. These behaviors were similar to those observed for the reported poly(acrylate) gel containing a thiourea group [22], and due to that, the thiourea moiety on the polysiloxane gel can bind the specific anions, such as acetate and fluoride, through hydrogen bond formation.

**Figure 7:**
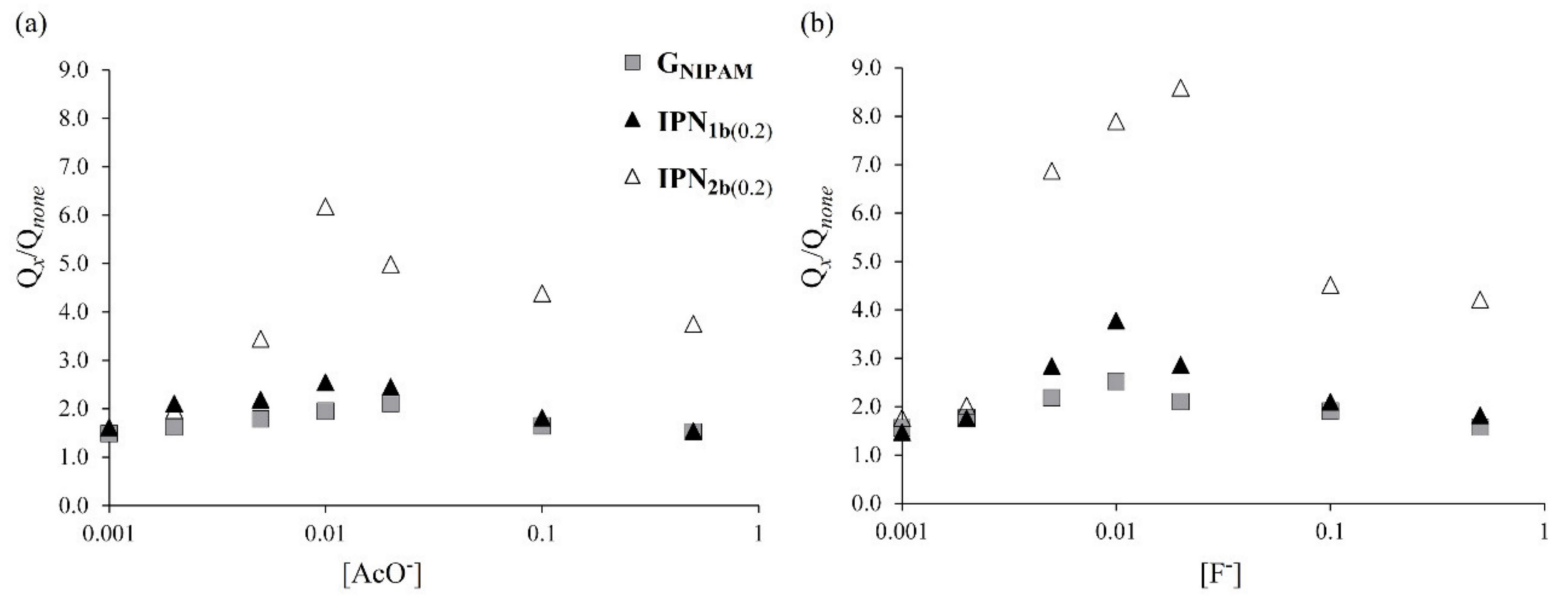
Log plots of relative swelling degree ratio (*Q*_x_/*Q*_none_) of **G*_NIPAM_*** (Table 2, run 1), **IPN*_1b(0.2)_*** (run 6), and **IPN*_2b(0.2)_*** (run 10) vs. concentrations (mol/L) of (**a**) acetate and (**b**) fluoride anions in DMF (25 °C).

The concentration dependence of the relative swelling ratios for the **IPN*_2b_*** in the solution of the acetate and fluoride ions was examined, and the results are shown in Figure 7 together with those for **IPN*_1b_*** and **G*_NIPAM_***. The observed ratios rapidly increased with the increase in the anion concentration and showed a maximum of around 10 to 20 mM, then they gradually decreased to a value which is still higher than those observed for **IPN*_2b_*** in the absence of the ions, as well as **G*_NIPAM_***. The observed decrease in *Q*_x_/*Q*_none_ at the higher concentration of the anions may be due to the increase in the polarity of the media [22]. In contrast, the **G*_NIPAM_*** barely showed a volume change, whereas a slight increase was observed for **IPN*_1b_*** based on the experiments changing the concentration of the fluoride ion.

## 4. Conclusions

Novel IPNs composed of PNIPAM and the silicone polymer containing urea and thiourea side groups were easily synthesized through the simultaneous reactions, namely, radical gelation and condensation of silanols on the silicone polymers. The obtained IPNs showed multi-responsive functions to the temperature based on VPTT in water and the chemical stimuli of acetate and fluoride ions in DMF, along with characteristic dual changes, such as color based on the *p*-nitrophenyl chromophores and volume due to the thiourea groups.

## Figures and Tables

**Table 1:** Preparation of **PSOL*_1_*** and **PSOL*_2_***.

Run	Silylation Reagent (Equiv.)		PSO-TMS	PSOL	
			Unit Ratio (R/MeO/TMS) ^1^	Abbreviation	Silanol Ratio (%) ^2^
1	**1a**	0.1	59/2/39	**PSOL*_1a(0.1)_***	18
2		0.2	65/3/32	**PSOL*_1a(0.2)_***	14
3		0.4	69/2/29	**PSOL*_1a(0.4)_***	12
4	**1b**	0.1	62/2/36	**PSOL*_1b(0.1)_***	16
5		0.2	64/1/35	**PSOL*_1b(0.2)_***	15
6	**2a**	0.1	57/3/40	**PSOL*_2a(0.1)_***	19
7		0.2	60/3/37	**PSOL*_2a(0.2)_***	17
8	**2b**	0.1	52/3/45	**PSOL*_2b(0.1)_***	23
9		0.2	54/2/44	**PSOL*_2b(0.2)_***	22

Conditions: **PSOL**/THF = 2 mL (theoretical concentration: 1.6 M). ^1^ Determined by ^1^H NMR analysis of the polymers (**PSO*_1_***-TMS and **PSO*_2_***-TMS) obtained by trimethylsilylation with an excess amount of TMSCl to **PSOL*_1_*** and **PSOL*_2_*** [12] (in CDCl_3_). ^2^ Calculated from the unit ratio of **PSO**-TMS, assuming that the RSi(OMe)_2_ unit is negligible.

**Table 2:** IPN synthesis with **PSOL*_1_*** and **PSOL*_2_***.

Run	Silicone Polymer	Gel Abbreviation	Yield (g) ^1^	*Q* ^2^
1	none	**G*_NIPAM_***	0.72	13.0
2	**PSOL*_1a(0.1)_***	**IPN*_1a(0.1)_***	0.75	9.9
3	**PSOL*_1a(0.2)_***	**IPN*_1a(0.2)_***	0.81	7.6
4	**PSOL*_1a(0.4)_***	**IPN*_1a(0.4)_***	0.84	7.0
5	**PSOL*_1b(0.1)_***	**IPN*_1b(0.1)_***	0.75	6.2
6	**PSOL*_1b(0.2)_***	**IPN*_1b(0.2)_***	0.68	3.9
7	**PSOL*_2a(0.1)_***	**IPN*_2a(0.1)_***	0.83	8.8
8	**PSOL*_2a(0.2)_***	**IPN*_2a(0.2)_***	0.83	10.4
9	**PSOL*_2b(0.1)_***	**IPN*_2b(0.1)_***	0.76	6.3
10	**PSOL*_2b(0.2)_***	**IPN*_2b(0.2)_***	0.69	5.5

Conditions: **PSOL**/THF = 2 mL (theoretical concentration: 1.6 M), NIPAM = 0.77 g, (NIPAM)/(BIS)/(AIBN) = 100/4/1, temp. = 60 °C, time = 24 h. ^1^ The obtained gel was immersed in THF for 2 days and in distilled water for 2 days. ^2^ In water at 3 °C. BIS: *N,N′*-methylenebisacrylamide; AIBN: azobisisobutyronitrile.

**Table 3:** *Q* values estimated for the obtained gels in the absence and presence of tetrabutylammonium salts (TBAX) in DMF.

X^−^	*Q*_x_			
	G*_NIPAM_*	IPN*_1b(0.2)_*	IPN*_2a(0.2)_*	IPN*_2b(0.2)_*
none	8.0	8.7	5.3	4.2
F^−^	17.7	24.9	30.5	36.1
Cl^−^	9.4	13.6	8.5	7.1
Br^−^	8.3	10.7	5.6	5.1
I^−^	8.0	8.5	6.7	4.0
CH_3_CO_2_^−^	16.8	21.3	19.5	20.9
NO_3_^−^	7.5	8.1	5.1	5.3
HSO_4_^−^	8.6	11.2	7.9	5.0

Conditions: TBAX = 20 mM, temp. = 25 °C.
